# Cesarean section on a rise—Does advanced maternal age explain the increase? A population register-based study

**DOI:** 10.1371/journal.pone.0210655

**Published:** 2019-01-24

**Authors:** Eva Rydahl, Eugene Declercq, Mette Juhl, Rikke Damkjær Maimburg

**Affiliations:** 1 Department of Midwifery, University College Copenhagen, Copenhagen, Denmark; 2 Department of Clinical Medicine, Aarhus University, Aarhus, Denmark; 3 Department of Community Health Sciences, Boston University School of Public Health, Boston, Massachusetts, United States of America; 4 Department of Midwifery, University College Copenhagen, Copenhagen, Denmark; 5 Department of Clinical Medicine, Aarhus University, Aarhus, Denmark; 6 Department of Obstetrics and Gynaecology, Aarhus University Hospital, Aarhus, Denmark; Federal University of Sergipe, BRAZIL

## Abstract

**Background:**

In Denmark, the cesarean section rate has increased by 49% between 1998 and 2015 and accounts for 21% of all births. Cesarean sections may cause short- as well as long-term consequences for both the mother and the child and impose further risks in future pregnancies. Delaying pregnancy until advanced maternal age at childbirth has been suggested as contributing to the increase. The proportion of women giving birth at 35 years or above increased from 15% (1998) to 21% (2015). Advanced maternal age at childbirth has been found to be related to increased pre-pregnancy morbidity and associated risk factors that may contribute to an increased risk of cesarean section. The aim of this study was to examine the association between advanced maternal age and cesarean section in a Danish population and the influence of demographic, anthropometric, health, and obstetric factors on this association.

**Methods:**

This study draws on a national population-based cohort study of all Danish births between 1998 and 2015 (N = 1,122,964). Maternal age less than 30 years serves as reference with the following age categories: (30–34 years); (35–39 years), and (40 years and above). The primary outcome was a cesarean section. Multivariate regression models with adjustment for demographic, health, pregnancy, fetal, and obstetric characteristics were performed with the results further stratified by parity.

**Results:**

In general, a positive association between advanced maternal age and cesarean section was found. Only minor changes in the risk estimate occurred after adjustment for relevant confounders. In comparison with the reference category, nulliparous women aged 35-39- years had twice the risk for cesarean section (adjusted odds ratio (AOR) 2.18, 95% confidence interval (CI) [2.11–2.26]) whereas for women of 40 years or above, the risk was more than tripled (AOR 3.64, 95% CI [3.41–3.90]). For multiparous women aged 35-39-years the risk was more moderate, but still with an AOR of 1.56, 95% CI [1.53–1.60], and for those 40 years and above, the AOR was 2.02, 95% CI [1.92–2.09].

**Conclusions:**

Overall, cesarean section increased with increasing maternal age. Adjustment for maternal and obstetric risk factors had only a minor influence on the association. The association was stronger in nulliparous women compared to multiparous women. Given the lack of impact of demographic and health risks on the relationship between maternal age and cesarean section, the authors suggest obstetric culture could be added to the list of risk factors for a cesarean. Future research on obstetric culture is recommended as are studies on a possible age-related decrease in the ability to maintain the progression of labor.

**Trial registration:**

The study uses depersonalized register data and has been approved by the Danish Data Protection Agency (2015-41-4168).

## Introduction

The worldwide increase in the use of obstetric interventions since the 1970s is a present cause of concern as interventions may not only reduce morbidity and mortality but also impose risks of adverse events or further interventions [1]. One of the most intrusive interventions, cesarean section (CS), has become increasingly common in most of the industrialized world [2]. The World Health Organization (WHO) and Organisation for Economic Co-operation and Development (OECD) have raised concerns regarding the frequent and increasing use of CS [1,2]. There is a lack of consensus concerning an appropriate CS rate. However, studies taking into account the variation between countries regarding e.g level of poverty, education, life expectancy measured in the Human Development Index (HDI-index) suggests a CS rate between 9–16% as an appropriate rate [3,4]. A cross-national study of 194 countries, using neonatal and maternal mortality as outcomes found 19% as an appropriate CS threshold which may be overestimated as no adjustment for HDI index was conducted [5]. Extensive uterine surgery, such as CS, is associated with short- and long-term risks that may extend years beyond the current birth and may affect the health of both the woman, her child, and future pregnancies [2].

The reasons for the increase in CS are multifactorial, but existing literature suggests that the increase is predominantly a result of advanced maternal age, particularly in nulliparous women [6]. In most of the industrialized world, social, educational and demographic changes have led to an increasing number of women postponing their pregnancies until late in their fertile life [7]. This social trend combined with the accessibility to birth control and infertility treatment has increased the proportion of women experiencing their first pregnancy after 35 years of age [7–10]. In Denmark, the proportion of nulliparous women giving birth at the age of 35 or above has increased from 6.7% in 1998 to 12.3% in 2015 [8]. Most pregnant women with advanced maternal age are initially healthy, have higher socioeconomic status than younger mothers, are multiparous and have a low obstetric risk profile. Likewise, older mothers are more likely to have a healthy lifestyle prior to and during pregnancy compared to younger women [10–13]. Research studies have consistently found an association between the increase in maternal age and an increase in CS [14,15]. As morbidity tends to increase with age, any group of women with advanced maternal age will include more individuals with age-related prenatal risk factors such as hypertension [7,16] diabetes mellitus [10] and high Body Mass Index (BMI) compared with a similar group of younger pregnant women [17,18]. Furthermore, studies have found that more women in the advanced age-group develop pregnancy-related complications including gestational diabetes [19], preeclampsia, and placenta previa [7,20,21].

Increased maternal age *per se* implies a deterioration of physiological functions including the genital tract, the uterine musculature, and the hormonal system [7]. There is evidence to support a continuous age-related decline in the physical ability to perform sufficient uterine contractions increasing a potential risk of labor dystocia [22,23]. Hence, the higher CS rate may also be a consequence of concomitant comorbidities related to advanced maternal age [15,24].

A more proactive obstetric practice has also been suggested to influence the increased CS rate. Interventions in pregnancy and childbirth have been on a rise cross-nationally [25]. The medicalization of pregnancy and childbirth causes concern as interventions developed for use in response to specific medical indications, may become harmful when used routinely due to the inherent risk of adverse effects [26]. The increased use of interventions in labor, including induction of labor, epidural analgesia, and continuous fetal monitoring, is associated with cesarean section [27–30]. Cesarean sections can be either planned during pregnancy or in response to events in labor. In Denmark, there has been a change in the use of planned CS. It was introduced as an option for coding in Danish obstetrics in 2000 and has since increased to 9% of all births in 2015 [8]. Planned CS may be optioned by the obstetrician or based on a request from a mother. There is also a substantial variation in overall CS rates according to geographical and birth setting factors. For example, in the US, rates vary from 7% to 70% between individual hospitals even after adjusting for maternal risk, suggesting that practice patterns may play a significant role in variation in delivery mode [31].

A lower threshold is suggested to influence the choice to perform CS among clinicians when treating women at advanced maternal age compared to younger age groups [6]. A Canadian study found that women with advanced age were more likely to be offered a CS or to ask for it themselves [10]. The challenge for researchers is to disentangle the impact of anxiety and subjective sense of urgency among mothers and the varying obstetric threshold for intervention that may influence the decision to perform a cesarean [16,32,33].

The aim of this study was to examine the association between advanced maternal age and CS in a Danish population and the influence of demographic, anthropometric, health, and obstetric factors on this association.

## Material and methods

This study examines the association between advanced maternal age at birth and cesarean section in a Danish population. We conducted a retrospective cohort study based on prospectively collected data from the Danish Medical Birth Registry (DMBR). This holds information on all births of women with a Danish civil registration number (CRN) or a temporary CNR and their live born and stillborn offspring. Illegal immigrants are likely to have their births obtained in the register, as they normally give birth anonymously within institutions. The dataset contains information on maternal demographic characteristics, ethnicity, educational and civil status, pathology and death. While they are part of the Danish Realm, data from Greenland and Faroe Island are not included. We included all deliveries recorded between 1 January 1998 and 31 December 2015 (N = 1,122,964). Descriptive statistics are given for the total study population, while regression analyses were stratified by parity and thus restricted to births with complete information on parity. The only exclusion criteria was cases with missing information on parity. We excluded 34,881 births with no information on parity, leaving 1,088,083 eligible for regression analysis (97%).

The dataset for this study (MIPAC, reference number 705026) is hosted by Statistics Denmark and draws on information from several additional national registers: The Danish Medical Birth Registry, The Danish National Diabetes Register, The Danish Register of Causes of Death, The Danish Pathology Register, and The Danish National Patient Register. Data were collected prospectively for the periods of pregnancy and childbirth. Throughout pregnancy, medical staff, primary midwives and obstetricians update information at all antenatal visits [34].

The exposure variable of interest was the maternal age at the time of birth. Maternal age was categorized into four age groups: <30 years; 30–35 years; 35-39-years and 40 years and above. The term “advanced maternal age” usually refers to a maternal age of 35 years or beyond according to existing literature [20].

The outcome variable of interest was overall CS, including both emergency and planned CS as classified by the International Classification of Diseases (ICD10).

*Maternal demographic characteristics* included marital status (”married/registered partnership”, yes/no); year of birth; citizenship (“Danish”,”Western” and “Non-Western”) and level of education (six levels ranging from “basic education” to “higher education /Ph.D”). *Maternal pregnancy characteristics* included parity; multiple gestations; previous cesarean; preeclampsia; placenta previa, and gestational age (“<37 weeks”, “37+0–41+6 weeks” and “42+0 weeks”). *Maternal health characteristics* included BMI (categorized according to WHO); smoking habits (“non-smoking” when not smoking or quitting in 1.trimester); hypertension; diabetes mellitus, and medical diagnosis prior to pregnancy. *Obstetric characteristics* included hospital size ("<1500", "1500–3000", "3000+" annual deliveries), use of epidural analgesia and induction of labor on an unfavorable cervix (prostaglandins or Foley catheter). *Neonatal characteristics* included birthweight (“<2500”, “2500–4000” and “>4000 grams at birth”); fetal presentation (vertex/ non-vertex), and stillbirth (yes/ no). See S1 Appendix for further details.

### Statistical analysis

Descriptive statistics were used to calculate absolute numbers and percentages on baseline characteristics for the total population and with stratification by maternal age-groups. Outliers were coded as missing values if a substantial gap was found in the tails of the normal distribution.

For births with complete information on parity, the association between age and CS was analyzed in univariate and multivariate logistic regression models and presented by AOR and 95% confidence intervals (CI). The multiple logistic regression model was built stepwise analyzing the impact of demographic, pregnancy, health, obstetric, and fetal factors on the association between advanced maternal age and CS.

Maternal age less than 30 years served as reference category due to their lower risk of comorbidities. The analysis was further stratified by parity (nulliparous vs multiparous women), as parity often acts as an effect modifier on the association between age and CS.

Binary variables were generated for respectively hypertension and diabetes, with variables representing cases of either preexisting or pregnancy-induced cases.

Tests for interaction between year and each of the included covariates were conducted, as changes over the 18-year study period may have influenced the results.

Given that the study population was more than 1,000,000 women, tests of statistical significance are not generally presented as even the smallest differences were significant. In order to include only relevant confounders in the final model, we excluded variables that showed no clinically relevant association in the bivariate analysis according to the authors. This excluded smoking. A post-estimation test for multi-collinearity was performed, and confounders were excluded if *P*-value >0.5 between variables. This excluded birthweight, which was correlated to gestational age.

The final model is presented in 6 steps with increasing adjustment levels: Step 1 was adjusted for year of birth. Step 2 added demographic factors (citizenship, education and marital status). Step 3 added pregnancy characteristics (multiple gestations, gestational age, severe preeclampsia, and placenta previa, and, for multiparous women, also previous CS). Step 4 added maternal health characteristics (hypertension, diabetes and other medical risks). Step 5 added obstetric related factors (induction of labor, epidural analgesia, hospital size), and, finally, step 6 added fetal factors (fetal presentation). Information on BMI was not implemented until 2003 and only consistent from 2004. Sub-analyses with and without BMI were performed on data from 2004–2015.

Data were analyzed in STATA/SE 15.1 software package (StataCorp. 2017. Stata Statistical Software). All reported *P*-values are two-sided, and the level of statistical significance was 5%.

## Results

During the 18-year study period, 1,122,964 births with a gestational age of 22 weeks or more were registered in Denmark. The majority of the women contributing with births to the study were multiparous (55%), the largest age group was 30–34 years (36%), 86% had a BMI less than 30, and 13% reported smoking during pregnancy.

### Description of population

The total population had a mean maternal age of 30.0 (SD 4.9) years ranging from 13 to 61 years. Stratified by parity, the mean age of nulliparous women was 28,2 (SD 4.8) years and of multiparous 31,5 years (SD 4.5).

Compared with the youngest age category, women 35 and older were more often multiparous, married, with a higher level of education, and did more often experience comorbidities or pregnancy-related conditions, such as placenta previa. Increased maternal age was associated with an increased likelihood of multiple gestations, hypertension during pregnancy, diabetes mellitus, gestational diabetes, and other medical diseases (Table 1). Mothers 35 or older were less likely to smoke during pregnancy and had a similar risk of being obese and develop severe preeclampsia as the youngest category. They were more likely to give birth at a larger birth unit (in Denmark, typically a tertiary hospital). They were more likely to have their labor induced, but less likely to have epidural analgesia for pain-relief. The offspring of women at maternal age 35 and above more often had a fetal presentation other than the vertex. The neonatal outcomes showed a generally slightly increased risk of Apgar <7 at 5 minutes, low birth weight, and stillbirth among women 40 years or above (Table 1).

**Table 1:** Maternal and fetal baseline characteristics among all Danish Births, 1998–2015 (n = 1,122,964).

Total number, n = 1,122,964				
**Maternal age, year**	**<30**	**30–34**	**35–39**	**40+**
	n = 517,450	n = 398,873	n = 175,280	n = 31,361
**Maternal demographics**				
***Citizenship***				
Danish	413,421 (84)	338,479 (88)	146,033 (86)	24,728 (82)
Western country	14,463 (3.0)	14,050 (3.6)	7,241 (4.3)	1,363 (4.5)
Non-western	61,642 (13)	33,464 (8.7)	16,895 (10)	4,145 (14)
***Educational level***				
Basic education	132,628 (28)	41,092 (11)	19,493 (12)	4,420 (15)
Secondary education	70,050 (15)	28,218 (7.5)	12,283 (7.4)	2,265 (7.8)
Skilled worker	149,859 (32)	112,339 (30)	45,359 (28)	7,264 (25)
Short higher education	18,063 (3.8)	22,677 (6.0)	9,668 (5.8)	1,515 (5.2)
Medium higher education	84,955 (18)	112,308 (30)	47,616 (29)	8,159 (28)
Long higher education	16,781 (3.6)	59,127 (16)	31,039 (19)	5,519 (19)
***Marital status***				
Married/registered	186,813 (36)	225,086 (56)	102,098 (58)	17,122 (55)
**Maternal pregnancy characteristics**				
***Parity***				
Nullipara	303,910 (61)	133,695 (34)	41,491 (24)	7,137 (23)
Multipara	196,501 (39)	254,677 (66)	128,901 (76)	23,280 (77)
***Multiple gestations***	7,201 (1.4)	9,007 (2.3)	4,856 (2.8)	942 (3.1)
***Gestational age***				
<37 weeks	31,897 (6.3)	22,644 (5.8)	11,247 (6.6)	2,445 (8.0)
37+0–41+6 weeks	446,401 (88)	346,498 (88)	150,916 (88)	26,688 (87)
42 + weeks	29,529 (5.8)	22,553 (5.8)	9,346 (5.5)	1,472 (4.8)
***Previous cesarean (mulipara)***	25,693 (13)	43,211 (17)	25,701 (20)	4,887 (21)
***Preeclampsia***				
Light/moderate	12,402 (2.4)	7,667 (1.9)	3,715 (2.1)	850 (2.7)
Severe	3,416 (0.7)	2,470 (0.6)	1,189 (0.7)	310 (1.0)
***Placenta previa***	1,929 (0.4)	2,462 (0.6)	1,600 (0.9)	408 (1.3)
**Maternal health characteristics**				
***BMI (2004–2015)***				
Underweight (<18,5)	15,945 (5.3)	9,114 (3.7)	3,444 (3.0)	558 (2.7)
Normal (18,5–24,9)	183,293 (61)	161,190 (64)	72,324 (63)	13,165 (60)
Overweight (25–29,9)	62,981 (21)	51,536 (21)	25,280 (22)	5,216 (24)
Obese (30+)	38,482 (13)	28,489 (11)	14,138 (12)	3,045 (14)
***Smoking***	85,399 (17)	41,787 (11)	19,850 (12)	3,653 (12)
***Hypertension***				
Before pregnancy	2,280 (0.4)	2,760 (0.7)	1,984 (1.1)	614 (2.0)
Pregnancy induced	6,985 (1.4)	6,105 (1.5)	3,482 (2.0)	948 (3.0)
***Diabetes***				
Established	2,555 (0.5)	2,503 (0.6)	1,553 (0.9)	425 (1.4)
Gestational	7,835 (1.5)	8,921 (2.2)	6,097 (3.5)	1,746 (5.6)
Medical risk prior to pregnancy*				
Yes	12,430 (2.4)	11,740 (2.9)	7,105 (4.0)	1,763 (5.6)
**Obstetric characteristics**				
***Hospital size (average 1998–2015)***				
<1500 annual deliveries	100,116 (20)	63,318 (16)	24,141 (14)	3,643 (12)
1500–3000	199,946 (40)	150,832 (39)	65,377 (38)	11,352 (37)
3000+	205,494 (41)	175,623 (45)	81,189 (48)	15,479 (51)
***Epidural use***	73,971 (14)	48,075 (12)	20,675 (12)	3,931 (13)
***Induction prostaglandin/foley***	55,758 (11)	43,790 (11)	21,944 (13)	5,307 (17)
**Neonatal characteristics**				
***Apgar score <7/5 min (%)***	3,885 (0.8)	2,848 (0.8)	1,425 (0.8)	309 (1.1)
***Birthweight <2500 gr***	22,816 (4.5)	16,865 (4.3)	8,868 (5.2)	2,014 (6.6)
**Fetal presentation**				
Other than vertex	20,868 (4.2)	17,530 (4,5)	8,463 (5.0)	1,716 (5.7)
***Liveborn*** **				
Not liveborn	1,632 (0.3)	1,300 (0.3)	705 (0.4)	191 (0.6)

Abbreviation: BMI, body mass index, Numbers may not sum to column totals because of missing values,

*Medical risks that can affect pregnancy and childbirth, Obtained from the medical record,

**Liveborn: excluded homebirth due to problematic coding on fetal death (n = 13,778 excluded), Missing variables do not extend 5% except from educational level (7%) and BMI (6%).

### The distribution of cesarean sections

**Fig 1:**
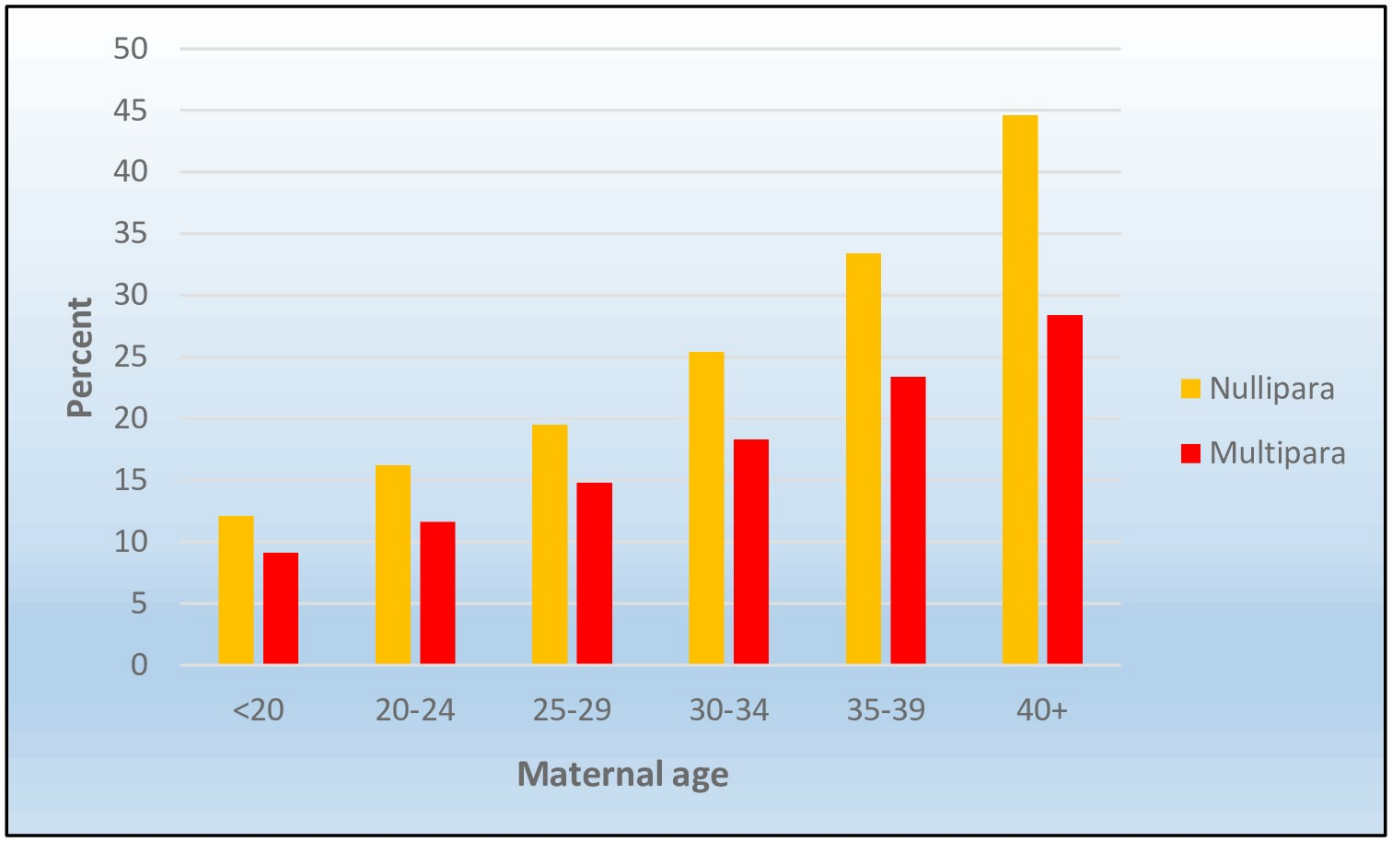
Unadjusted risk of cesarean section stratified by parity and age (years).

The proportion of women giving birth by CS increased with increasing maternal age. Nulliparous women had a higher risk of CS than multiparous women in all age categories (Fig 1). For nulliparous women, the risk of CS almost quadrupled from the youngest to the oldest age category (from 12% to 45%). A similar, though less pronounced pattern was observed among multiparous women, with a three-fold increase (from 9% to 28%) (for absolute numbers, see S2 Appendix).

**Fig 2:**
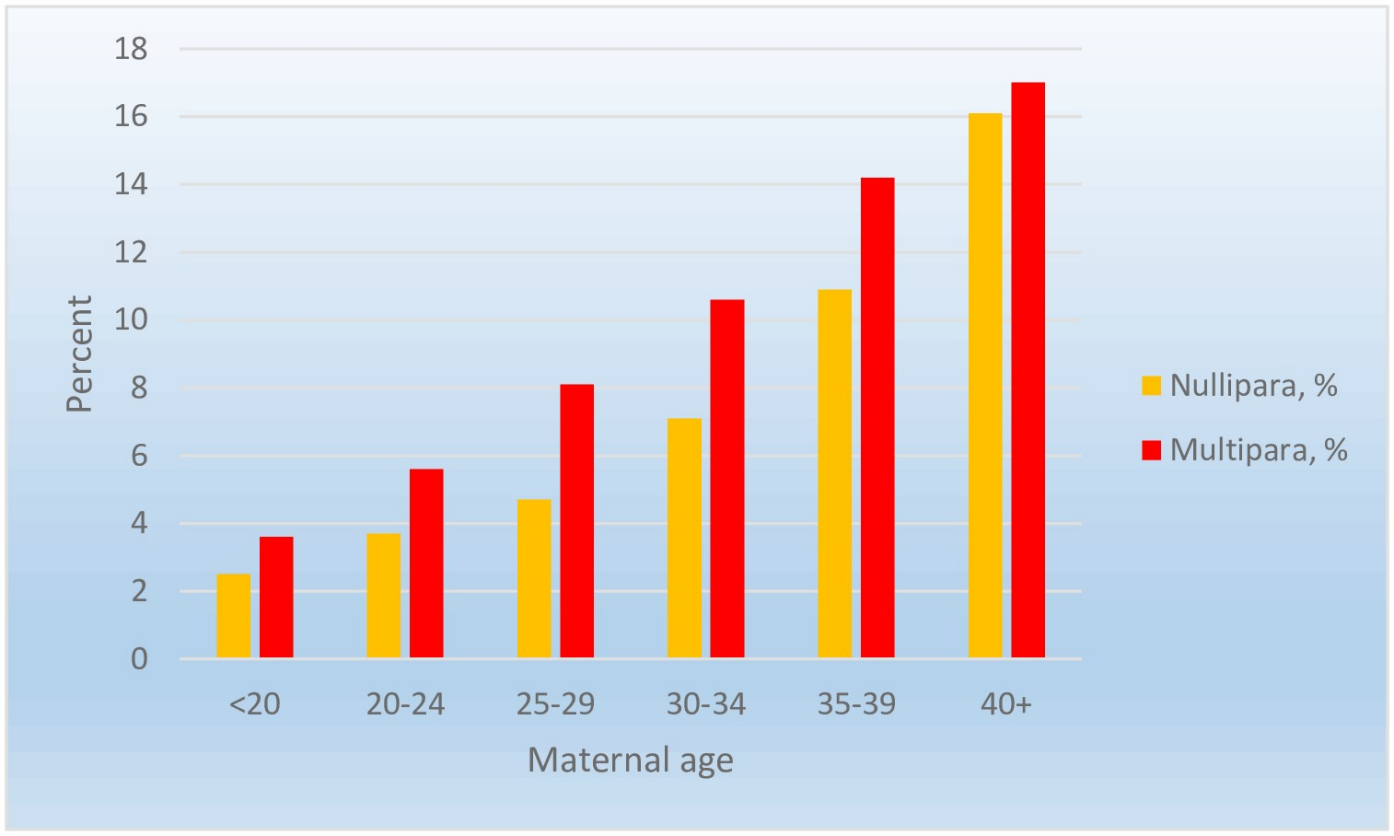
Unadjusted risk of planned cesarean section. Stratified by parity and age (years).

Fig 2 shows the association between maternal age and planned CS by parity. Planned CS varied substantially from younger to older age, ranging from 2–3% among women younger than 25 years to about 16% among women aged 40 years or above (for absolute numbers, see S2 Appendix).

Tables 2 and 3 present Adjusted Odds Ratios for the association between age and overall CS with a stepwise inclusion of additional sets of confounders for nulliparous and multiparous women, respectively. An increased risk of CS with increasing maternal age was consistent for both nulliparous and multiparous women, though more pronounced for the former. Adding possible confounding variables to the regression model for nulliparous women only made small changes in risk estimates. All single confounders were added separately in the model. No single confounder changed the results significantly and none of the confounders outweighed the other confounders. Only adding previous CS to the model changed the result for multiparous women, attenuating the association between age and CS. In the full regression model for nulliparous mothers age 40 years and above, the risk estimate changed from an unadjusted OR 3.63, 95% CI [3.46–3.81] to an adjusted OR (AOR) of 3.64. 95% CI [3.41–3.90]. Among multiparous women age 40 and above there was a change, from an unadjusted OR of 2.39, 95% CI [2.32–2.47] to an AOR 2.02, 95% CI [1.92–2.09] with the shift occurring after the addition of the pregnancy characteristics. The sub-analysis including BMI from the study years 2004–2015 did not notably change the results.

**Table 2:** Nulliparous women. Adjusted Odds Ratio (AOR) with 95% Confidence Interval (CI) of cesarean section. Women < 30 years are used as the reference category.

	Nulliparous, n = 485,469			
	Ref <30 yrs	30–34 yrs.	35–39 yrs.	40+ yrs.
	n = 303,433	n = 133,485	n = 41,430	n = 7,121
Unadjusted	1 (ref.)	1.53 (1.51–1.56)	2.26 (2.21–2.31)	3.63 (3.46–3.81)
Adjusted for year	1 (ref.)	1.53 (1.50–1.55)	2.23 (2.18–2.28)	3.56 (3.39–3.73)
Adjusted for demographics ^a^	1 (ref.)	1.57 (1.55–1.60)	2.31 (2.26–2.37)	3.65 (3.46–3.84)
Adjusted for pregnancy characteristics ^b^	1 (ref.)	1.52 (1.50–1.55)	2.18 (2.12–2.23)	3.45 (3.27–3.63)
Adjusted for health characteristics^c^	1 (ref.)	1.50 (1.48–1.53)	2.11 (2.06–2.16)	3.25 (3.09–3.44)
Adjusted for obstetrics ^d^	1 (ref.)	1.50 (1.47–1.52)	2.10 (2.05–2.16)	3.23 (3.06–3.41)
Adjusted for fetal factors ^e^	1 (ref.)	1.53 (1.49–1.57)*	2.18 (2.11–2.26)*	3.64 (3.41–3.90)*
Subanalysis adjusted for BMI class, only data from 2004–14				
Adjustment for BMI	1 (ref.)	1.52 (1.48–1.56)	2.17 (2.10–2.25)	3.69 (3.44–3.95)

^a:^ marital status, citizenship, education

^b:^ multiple gestations, gestational age, preeclampsia, placenta previa

^c:^ hypertension, diabetes, other medical diseases

^d:^ hospital size, epidural, induction of labor

^e:^ fetal presentation

*: P-values <0.0001

**Table 3:** Multiparous women. Adjusted Odds Ratio (AOR) with 95% Confidence Interval (CI) of cesarean section. Women < 30 years are used as the reference category.

	Multiparous, n = 602,614			
	Ref <30 yrs	30–34 yrs.	35–39 yrs.	40+ yrs.
	n = 196,229	n = 254,396	n = 128,744	n = 23,245
Unadjusted	1 (ref.)	1.35 (1.33–1.37)	1.85 (1.81–1.88)	2.39 (2.32–2.47)
Adjusted for year	1 (ref.)	1.33 (1.31–1.35)	1.78 (1.75–1.82)	2.27 (2.20–2.34)
Adjusted for demographics^a^	1 (ref.)	1.39 (1.36–1.41)	1.89 (1.85–1.92)	2.39 (2.31–2.47)
Adjusted for pregnancy characteristics^b^	1 (ref.)	1.26 (1.23–1.28)	1.63 (1.59–1.66)	2.10 (2.02–2.19)
Adjusted for health characteristics^c^	1 (ref.)	1.24 (1.22–1.27)	1.59 (1.55–1.63)	2.02 (1.94–2.10)
Adjusted for obstetrics ^d^	1 (ref.)	1.23 (1.21–1.26)	1.57 (1.54–1.61)	2.02 (1.94–2.10)
Adjusted for fetal factors ^e^	1 (ref.)	1.24 (1.21–1.26)*	1.56 (1.53–1.60)*	2.02 (1.92–2.09)*
Subanalysis adjusted for BMI class, only data from 2004–14				
Adjustment for BMI	1 (ref.)	1.21 (1.18–1.24)	1.49 (1.45–1.53)	1.92 (1.83–2.02)

^a:^ marital status, citizenship, education

^b:^ multiple gestations, gestational age, previous cesarean, preeclampsia, placenta previa

^c:^ hypertension, diabetes, other medical diseases

^d:^ hospital size, epidural, induction of labor

^e:^ fetal presentation

*: P-values <0.0001

## Discussion

### Principal findings

This study included more than 1.1 million Danish births over a time period of 18 years and showed a strong and consistent association between advanced maternal age and CS. The CS risk was increased in all age categories of 30 years or more compared to women younger than 30 years, and it was consistent after adjustment for maternal demographic and health-related factors. Among nulliparous women, the risk of CS was 3.6 times higher in women of 40 years or above compared to women less than 30 years. For multiparous women, the CS risk was doubled in women of 40 years or above and consistent after adjustment for previous CS.

### Strengths and weaknesses

Despite the comprehensive dataset, the study has limitations. The design does not allow for causal interpretation of the associations studied. The confounders in the adjusted models were restricted to those available in the registers. We did not have information regarding assisted reproductive technology (ART), which may be correlated to increased maternal age. We adjusted for multiple gestations and being born preterm. We considered both as surrogate measures in lack of better data, as both can be related to ART. Some studies adjust for health insurance [14,19], but in Denmark, all mothers are covered and have equal access to healthcare. Further, it is common to adjust for living in an urban or rural area. This is, however, unlikely to bias the results, as Denmark geographically is very condensed with the longest distance to a birth unit about 1 hour by car. Incomplete registration of diagnoses or other characteristics may impose unmeasured confounding. No recent validation of the registers has been performed, but a study from 2003 found that the most common surgical interventions, diagnoses, and procedures were valid when matched with medical records data [35]. The variables used in our study were based on ICD-10 main diagnosis categories, which have been found to be of high quality [35]. Also, interventions that are followed by reimbursement to the hospital have been found to be well-reported in the registers [36], which is the case for our outcome of interest, cesarean section.

A strength of this study is that it included the total population of women in Denmark giving birth during the study period, which makes selection bias unlikely. Selection bias is further minimized, as Denmark has a universal health care system and only a small private hospital sector, which allows for a collection of information from all hospitals, and women across all income levels and demographic characteristics. Linkage of data on an individual level and merging of several registers made it possible to include and combine demographic, social, and medical information. Data were collected and registered prospectively during pregnancy and childbirth at all contacts with midwives, general practitioners, obstetricians, and other healthcare professionals. Registration was controlled at the hospital level and at Danish National Patient Register for missing codes, errors in civil registration number, and incorrect digits [37]. In our study, the number of missing values were typically less than 5%. BMI had 6% missing values. BMI was only included in a sub-analysis from 2004–2015 as data were not collected before. Approximately 7% of women had missing values on the education variable. However, CS did not increase with increasing levels of education. Furthermore, the missing values for education were evenly distributed in all age groups, and therefore less likely to influence the risk estimate.

### Interpretation

Our results are consistent with existing literature reporting a significant association between advanced maternal age and CS [14,18,19,23,38]. The strength of the association, and the fact that other studies find similar results support the hypothesis of a causal relationship between maternal age and CS. Comprehensive adjustment for known confounders was conducted. By using this approach, we incorporated the effect of pre-pregnancy registered comorbidities, demographic and anthropometric factors as well as pregnancy, obstetric, and fetal complications. Stepwise regression showed that no single variable had a significant impact on the risk of CS by age in nulliparous women, while adjustment for pregnancy characteristics did have an influence on the association for multiparous women. Timofeev et al studied a US American population and performed nearly the same adjustments as in our study. They found similar results of increased risk of CS in nulliparous women aged 40–45 years (AOR 2.77, 95% [2.50–3.07]) compared to women ages 25–29 years [19]. Richards et al found an increase in primary CS with increased maternal age, regardless of parity [14]. Herstad et al identified a low-risk population of nulliparous women and found an increase in emergency CS with advanced maternal age [23].

A common explanation for the increased risk of CS with advanced maternal age is pre-pregnancy morbidities. The risk of many diseases has been shown to increase with increased maternal age. However, as noted in Table 1, comorbidities were only present in a minority of women in our study, whereas the large majority remained healthy, and the association between maternal age and CS were only slightly attenuated after adjustment. This is consistent with other studies [14,19,23,38]. What causes the strong association between advanced maternal age and risk of CS thus remains unexplained. In the search for other plausible explanatory etiologic factors, dysfunctional labor patterns decreased myometrial efficiency, and abnormal uterine implantations have been mentioned [14,27,33,38]. Others suggest the impact of birth culture and maternal preferences [27,31,33]. The latter is, however, difficult to measure in population databases, and associated variables were not available in our data and could therefore not be controlled for.

### A decrease in physical ability to perform

The evidence of a decreased ability for physical performance remains unclear. Our data are consistent with other studies regarding an increase in placenta previa and fetal mal-presentations with increased maternal age. In our study, the frequency of placenta previa was 0,4% among women younger than 30 years and 1,3% among women of 40+ years. A systematic review by Martinelli et al found similar results, even after stratifying for parity. The authors suggest that atherosclerotic changes in the blood vessels may impair the flow and infarction as underlying causes, which may provoke a lower implant of the placenta in the uterine cavity [39]. Regarding fetal presentation, we found a non-vertex presentation of the fetus in 4,2% of women younger than 20 years and 5,7% in 40+ -year-old women. This tendency is supported by Timofeev et al and may support the argument of change in the uterine performance [19]. Uterine scar and premature births are independent risk factors for non-vertex presentations [40]. Therefore, an increased likelihood of uterine scars and the slight increased risk of preterm birth among older women in our study may explain the higher frequency of non-vertex presentation.

Medical augmentation to offset labor dystocia is more frequently used among women at advanced maternal age compared to younger groups, suggesting a decreased ability for the uterus to perform sufficient progression during labor [22,23,38]. Waldenström et al found a doubled risk of labor dystocia in 35–39 year-old nulliparous women compared to women younger than 25 years (AOR 2.13 95% CI [2.06–2.20]). This tendency was consistent even stratified by the women’s first, second and third birth [22]. Herstad et al found similar results and found the association significantly reduced after exclusion of birth weight > 4000 grams, gestational age 42+0 weeks or above, and epidural analgesia, suggesting these conditions as contributing factors to labor dystocia [23]. Herstad et al also found dystocia to be the most frequent indication for acute CS [23].

Higher augmentation rates, and subsequent increased risk of CS, among women of older age may be caused by physical factors related to older age, as discussed above. However, the frequent use of labor augmentation in women of advanced maternal age may also be a result of cultural trends. It has been suggested that health professionals have a lower threshold for diagnosing this group with labor dystocia [23,38].

Labor dystocia is closely related to the length of labor, however, studies on the length of labor show conflicting results. Greenberg et al found longer first and second stages of labor in women of advanced age [41], while Zaki el al found labor progression to be more efficient in older women suggesting sufficient uterine ability to perform contractions [42]. The Zaki-study did not adjust for labor induction, labor augmentation, or epidural analgesia, which may have influenced their results. The authors, however, presented information on the fact that the use of these interventions did not differ between age categories [42]. Impaired myometrical contractility due to advanced maternal age may be clarified by in vitro testing. Crankshaw et al examined the efficiency of uterine contractility using biopsy samples from women aged 28–52 years and found no evidence of impairment in contractility of human myometrical tissue with increasing age [43].

### Medicalization

Over the last four decades, the concept of medicalization has become a field of interest, especially in social- science disciplines. It generally describes a process where non-medical problems become defined as medical problems and treated as such, and thus, conditions that are within the normal variation are being pathologized [44]. The process may be driven by new evidence or hypotheses, but also of potential influence are the attitudes of health-care personnel, the pharmaceutical industry, and maternal preferences [44]. Furthermore, it has been suggested that women are more susceptible to medicalization than men, which might also be reflected in the manner in which common life processes, such as birth control, menopause, and childbirth, have become medicalized [44,45]. Several studies suggest the increase in CS among women with advanced maternal age to be influenced by a lowered treatment threshold for interventions [10,18,23,38].

### A lowered threshold

According to Omih & Lindow, the fact that women at an advanced age are considered at high risk may have an iatrogenic impact on the provided care. The perception of risk may lower the threshold for interventions among obstetric personnel [38]. Gerli et al emphasize that the choice of performing a CS could more easily be made among older patients, as the pregnancy tends to be ‘precious’ and at ‘high risk for medical-legal issues’ [32]. A US study found a decrease in CS rates if the birth care provider was favorable toward vaginal birth [46]. A similar difference in threshold may be observed among different birth units [47]. A British study monitored the association between advanced maternal age and a composite measure of birth outcomes according to the birth setting. Women were more likely to have interventions if they gave birth in a specialized obstetric department compared to a midwifery-led care unit, and the association was more pronounced among women at advanced maternal age [47].

In the present study, we were unable to disentangle a possible influence of medicalization. However, the rate of planned CS may be an indicator of lower treatment threshold in birth care providers or of women’s preferences. Elective CS does not necessarily reflect biology but perhaps serves more as an indicator of care and preferences [18]. A British study of 51,225 women found an increase in planned CS from 2% to 22% in women age 20 to 40 years of age [18]. This is similar to our study, where the CS rate was 2–4% in the two youngest age-categories (less than 24 years) and 16–17% in the oldest group (at or above 40 years) with only marginal changes when stratified by parity. This may suggest a lower threshold towards planned CS among women at advanced maternal age.

### Women’s preferences

Limited evidence is available on women’s preferences as a driving force for lowering the threshold. Fox & Worts conducted in-depth interviews with 40 women on their transition to parenthood. They found that the women turned their concerns exclusively toward their child when monitoring processes indicated a problem. According to the authors, this may be the main reason why many women embrace medical management [45]. A US study on 1,960 women without previous CS examined the impact of anxiety in pregnancy by monitoring women with falsely suspected large babies. These women were more likely to be induced (AOR 1.9, 95% CI [1.4–2.6]), have an epidural (AOR 2.0, 95% CI [1.4–2.9]), or ask for an elective CS (AOR 4.6 95% CI [2.8–7.6]) than women without suspected large babies [48]. A study of 1.865 Canadian women found women at advanced maternal age (35 years and above) were twice as likely to request CS from their care provider during their pregnancy (OR 1.91, 95% CI [1.07 to 3.41]) compared to women aged 25–29 years [10]. DeVries argues that what women want in childbirth can both be constructed by and can itself construct the care, they receive [49].

It is thus possible that the articulation of high maternal age as being problematic has an impact on the choices made by the woman and can affect her confidence regarding her own abilities to give birth without the use of interventions. The present study cannot explain the robust association between increased age and CS, but it seems fair to suggest, that both culture and biological factors related to older age are possible important factors, that merit further investigation.

## Conclusion

The aim of this study was to analyze the association between advanced maternal age at birth and CS in a population-based study on 1,1 million Danish women. Results from this study supported the hypothesis of a strong association between increased maternal age and CS. The association could not be explained by demographic characteristics, maternal comorbidities, or complicated pregnancies. This leads the authors to add culture to the list of CS risk factors and encourage others to address this in future studies. Also, further research is needed on a possible age-related decrease in the ability to maintain the progression of labor. More knowledge should be generated on lowered treatment threshold as a provider attitude and/or a consequence of the women´s preferences in order to understand the distinct association between advanced maternal age and CS.

## Supporting information

S1 Appendix Classifications of variables. (DOCX)

S2 Appendix Cesarean section absolute numbers (%). (DOCX)

## Abbreviations

AOR: Adjusted Odds Ratio
BMI: Body Mass Index
CI: Confidence Interval
CS: Cesarean section
OR: Odds Ratio
WHO: World Health Organization
