# Supplementary material for: Cesarean section on a rise—Does advanced maternal age explain the increase? A population register-based study
Source: PLoS One. 2019 Jan 24;14(1):e0210655. doi: 10.1371/journal.pone.0210655 (PMC6345458; doi:10.1371/journal.pone.0210655)
Supplement: S1 Appendix — (DOCX) [file pone.0210655.s001.docx]

***S1 Appendix, Classifications of variables***

| **Name** | **System** | **Variable** |
| --- | --- | --- |
| Body Mass Index | WHO references | underweight (BMI <18,5), normal (BMI 18,5-24,9),  overweight (BMI 25-29,9), obese (BMI 30+) |
| Cesarean section, types | DNBR | **Planned:** planned CS on singleton or multiples. **Acute:** acute CS before or under labor. **Unspecific:** Type of CS not accounted for. In case of double coding, prelabor overrules. The only exception is when augmentation of labor have been used. Then codes are transformed to in labor cesaeran. |
| Citizenship | Statistics Denmark | **“Danish”** (Denmark, Greenland, Faroe Island), **“Western”** (EU, USA, Canada, New Zealand, Norway, Island, Lichtenstein, Monaco, Andorra, San Marino, The Vatican, Switzerland, Australia),  **“Non-western”** (remaining countries) |
| Diabetes | DNBR | **Chronic:** Preexisting type1 or type 2 or newly discovered but manifest Diabetes; **Gestational:** gestational Diabetes treated either with diet or with insulin. |
| Education | Statistics Denmark | Highest completed education. The six categories are done as suggested by Statistics Denmark |
| Epidural analgesia | DNBR | Epidural in cases, when registered as pain relief in vaginal birth or intended vaginal births. |
| Fetal presentation | DNBR | Dichotomized into vertex/ non-vertex |
| Hospital size | DNBR | Absolute number |
| Hypertension | DNBR | **Chronic**: Medically treated and/or diagnosed by an obstetrician before gestational week 20. **Gestational:** without protein in urin; Blood pressure >=140/90 repeatedly in rest or cases necessitating treatment. |
| Induction of labor | DNBR | Induction of labor on unfavorable cervix: Foley Catheter or prostaglandins / misoprostol |
| Marital status | Statistics Denmark | Yes: “married or in registered partnership” It is possible to have partnership registered for partners of same sex. |
| Medical risk prior to pregnancy | DNBR | The physician evaluate if the woman has a pre-existing medical condition yes/no |
| Multiple gestations | DNBR | Singleton or multiple gestation |
| Parity | DNBR | Primiparous or multiparous |
| Placenta Previa | DNBR | Yes / No |
| Preeclampsia | DNBR | **Light/ moderate:** Blood pressure >= 140/90 with proteinuria; **Severe***:* Blood pressure >=160 /110 with proteinuria and/or affected blood tests or subjective symptoms. |
| Smoking | DNBR | 0 "not smoking" 1 "smoking" |
| Stillbirth | DNBR | Liveborn at birth yes/no |

Abbreviations: DNBR: Danish National Birth Registry, WHO: World Health Organization

The used DNBR codes can be found in Danish: <http://www.dsog.dk/koder-og-kvalitetssikring/obstetriske-og-fotalmedicinske-koder/> under “Anbefalede obstetriske koder” [recommended obstetric codes]
