# Supplementary material for: Cesarean section on a rise—Does advanced maternal age explain the increase? A population register-based study
Source: PLoS One. 2019 Jan 24;14(1):e0210655. doi: 10.1371/journal.pone.0210655 (PMC6345458; doi:10.1371/journal.pone.0210655)
Supplement: S2 Appendix — (DOCX) [file pone.0210655.s002.docx]

***S2 Appendix, Cesarean section absolute numbers (%)***

| Total number, n=1,122,964 | | | | | | |
| --- | --- | --- | --- | --- | --- | --- |
| **Maternal age (year)** | **<20** | **20-24** | **25-29** | **30-34** | **35-39** | **40+** |
| **Cesaraen Section (all)** |  |  |  |  |  |  |
| Nullipara | 1,727 (12) | 14,210 (16) | 39,246 (20) | 33,963 (25) | 13,848 (33) | 3,179 (45) |
| Multipara | 113 (9) | 4,102 (12) | 23,661 (15) | 46,498 (18) | 30,130 (23) | 6,597 (28) |
|  | | | | | | |
| **Planned cesarean** |  |  |  |  |  |  |
| Nullipara | 356 (2.5) | 3,251 (3.7) | 9,537 (4.7) | 9,507 (7.1) | 4,521 (11) | 1,151 (16) |
| Multipara | 44 (3.6) | 1,986 (5.6) | 12,957 (8.1) | 27,078 (11) | 18,255 (14) | 3,960 (17) |
| Missings 3.1% | | | | | | |
